# Factors influencing the intention of young adults to adopt genotype-based personalised advice on diet and physical activity according to perceived weight status

**DOI:** 10.1017/jns.2024.50

**Published:** 2024-09-30

**Authors:** Alexandra King, Mark Glaister, Kate Lawrence, Leta Pilic, Yiannis Mavrommatis

**Affiliations:** 1 Faculty of Sport, Technology and Health Sciences, St Mary’s University, Twickenham, UK; 2 Optimyse Nutrition LTD, London, UK; 3 Lake Lucerne Institute, Vitznau, Switzerland

**Keywords:** Intention, Nutrigenomics, Personalised nutrition, Survey, Theory of planned behaviour

## Abstract

Genotype-based dietary and physical activity advice can be delivered to young adults before unhealthy lifestyle behaviours or metabolic and physiological conditions have developed. The aim of the present study was to investigate the factors that influence the intention to adopt genotype-based personalised advice on diet and physical activity in young adults who perceive themselves to be a healthy weight versus those who perceive themselves to be overweight or obese. An online survey of 396 young adults (18–25 years) evaluated background factors (participant characteristics (including perception of body weight), psychological factors, belief composites) and constructs of the Theory of Planned Behaviour (TPB) related to the adoption of genotype-based personalised advice. The association between background factors and TPB constructs was assessed using multiple linear regression. The constructs of TPB predicted intention to adopt genotype-based personalised nutrition (*P* < 0.001, adj. *R*^*2*^ = 0.54; attitude: *B* = 0.24, subjective norm: *B* = 0.25, PBC: *B* = 0.45). Background factors including belief composites, health locus of control, gender, physical activity, and food choice motives of ‘health’, ‘price’, ‘familiarity’, ‘weight control’, and ‘convenience’ significantly added to models of TPB constructs related to the intention to adopt personalised advice (*P* < 0.05). The influence of background factors varied between TPB constructs and differed based on participants perception of their body weight. The study provides support for the use of the TPB in understanding the intention of young adults to adopt gene-based advice for dietary and physical activity behaviour. In addition to perceived body weight, the background factors identified should help to inform and modify the delivery of advice in behaviour change interventions that seek to use genotype-based personalised advice in young adult populations.

## Introduction

Modification of lifestyle behaviours, including diet and physical activity, can considerably reduce the prevalence of non-communicable diseases (NCDs), reducing the burden of disease for both the individual and society.^(1)^ However, generic public health advice to address dietary and physical activity behaviours is not adhered to.^(2,3)^ Compared to this ‘one size fits all’ approach to dietary and physical activity advice, researchers have hypothesised that personalisation of advice based on an individual’s genotype could motivate greater adherence to guidance.^(4)^

Genotype-based personalised advice is usually delivered in combination with other levels of personalisation (phenotypic, clinical, dietary), with the aim to provide more precise and effective advice as well as to encourage behaviour change.^(5)^ Studies that have investigated the effect of genotype-based dietary advice on behaviour change have reported contradictory findings, both within and between studies.^(6–9)^ Recent systematic reviews and a meta-analysis of studies that have investigated the effect of genotype-based advice to motivate dietary and physical activity behaviour have reported no beneficial effect above that seen with other levels of personalisation.^(10,11)^ However, one benefit of genotype-based personalisation of advice over other levels of personalisation is that it can be delivered earlier in the lifespan, before unhealthy lifestyle behaviours or metabolic and physiological conditions have developed. Therefore, young people stand to benefit the most from genotype-based dietary and physical activity advice.^(12,13)^ Furthermore, young people have been reported to be more likely to adopt personalised nutrition compared to other age groups.^(14)^ However, to effectively implement genotype-based personalised advice to affect behaviour in young adults, an understanding of factors that may encourage or prevent engagement is required.

Interventions designed to change health-related behaviours are more likely to be successful when theoretical links between the intervention and the behaviour have been considered in the design.^(12,15–17)^ One of the most frequently cited behaviour change theories, the Theory of Planned Behaviour (TPB),^(15,18)^ states that ‘intention’ to perform a behaviour can be predicted from three independent constructs: attitude toward the behaviour, subjective norms, and perceived behavioural control (PBC).^(18)^ ‘Attitude toward the behaviour’ represents the extent to which an individual has a favourable appraisal of that behaviour, ‘subjective norms’ is the individual’s perceived social pressure to perform or not perform the behaviour and ‘PBC’ is an individual’s perception of how easy or difficult it is to perform the behaviour.^(19)^ Each construct of the TPB is influenced by belief composites: behavioural beliefs, normative beliefs, and control beliefs.^(18)^ Intention and PBC have been demonstrated to account for a significant amount of variation in numerous health-related behaviours including food choice, multiple correlations ranging from 0.20 to 0.78.^(18,20)^ Furthermore, background factors such as demographic characteristics, personality traits, and life values are reported to influence intention to perform a behaviour by affecting TPB constructs.^(19)^ There are several background factors that previous research has identified that may influence intention to engage with personalised advice: optimistic bias, the phenomenon by which an individual underestimates their own risk of developing a disease compared to others^(21)^; health locus of control (HLC), whether an individual perceives their health to be under their control (internal) or not (external)^(22)^; food choice motives, nine factors that have been shown to influence food choice^(23)^; and participant characteristics, such as sex and personal history of NCD.^(13,14,24–27)^

**Fig. 1.**
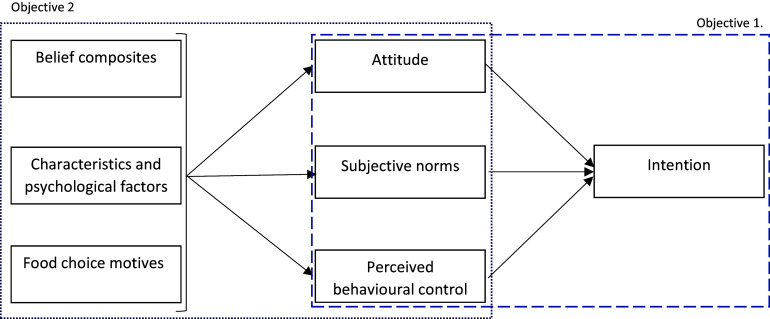
Specification of theory of planned behaviour model and study objectives. .

Although these factors have been associated previously with intention to engage with personalised advice, an understanding of how TPB constructs, belief composites, and background factors relate to the intention to adopt genotype-based personalised nutrition specifically in young adults has not been investigated. A clearer understanding of associations between these background factors and the intention to utilise personalised advice would inform researchers and health practitioners on how best to communicate advice to promote healthy lifestyle behaviours. Therefore, the aim of the present study was to investigate the factors that influence the intention to adopt genotype-based personalised advice for diet and physical activity in young adults; and to determine if factors differ in young adults that perceive themselves to be a healthy weight and those that perceive themselves to be overweight or obese. The overall aim was broken down into two objectives presented in Fig. 1.

## Methods

### Participants

A total of 414 responses were received for the survey, 18 were screened out due to not meeting the inclusion criteria. Therefore, 396 male and female young adults aged 18–25 years, living in the UK, who were not pregnant, lactating, following a restricted diet, or having a diagnosed eating disorder took part in the survey. Participants were recruited through advertisements shared during lectures at St Mary’s University and social media postings (Facebook, Twitter and LinkedIn). Data were collected between March and November 2022 using the Jisc online surveys platform (https://www.onlinesurveys.ac.uk) to ensure data are stored in a secure and GDPR-compliant environment.

### Survey development

A pilot survey was conducted in 35 young adults (18–25 years) to assess the usability of the survey and develop the TPB questionnaire.^(28)^ Items used to measure the TPB constructs were assessed for internal consistency^(29)^ and discriminant validity.^(30)^ To measure belief composites, free-response questions were used to elicit behavioural outcomes and experiences (perceived advantages, disadvantages and feelings), normative referents (individuals or groups that would approve or disapprove), and control factors (factors that would make it easy or difficult) in relation to the adoption of genotype-based advice to modify dietary or physical activity behaviour. Content analysis of free-response questions was used to construct items to be used in the final survey^(28)^ (Supplementary Material Tables 1–3).

### Final survey

The final survey was divided into three sections. The first section asked participants about characteristics: gender, age, ethnicity, education, perceived health, physical activity behaviour, and their perceived body image. Physical activity was assessed using a single question to determine whether participants were sufficiently active to benefit their health: ‘In the past week, on how many days have you done a total of 30 min or more of physical activity, which was enough to raise your breathing rate? This may include sport, exercise, and brisk walking or cycling for recreation or to get to and from places, but should not include housework or physical activity that may be part of your job’.^(31)^ To measure perceived body image, participants were asked to indicate their own body figure by choosing a silhouette of the Stunkard Scale. Based on the selected silhouette participants were classed to perceive themselves as underweight, normal weight, overweight or obese.^(32–34)^

The second section asked participants about their HLC, motives for food choice, and optimistic bias. For each scale, internal consistency was checked; Cronbach’s alpha (α) for all factors indicated adequate internal consistency.^(29)^ To assess HLC, participants were asked to indicate the extent to which they agreed or disagreed with six statements. For example: ‘I can be as healthy as I want to be.’ Response: Completely disagree, Disagree, Neither disagree/nor agree, Agree, Completely agree.^(25,35)^ The internal HLC was calculated from the average score for the first three items (α = 0.77) and external HLC from the second three items (α = 0.70).^(35)^ Motives for food choice were measured using the Food Choice Questionnaire.^(23)^ The 36 items represent nine factors and the mean score from 1 to 5 was calculated for each factor (health (α = 0.86), mood (α = 0.88), convenience (α = 0.87), sensory appeal (α = 0.82), natural content (α = 0.88), price (α = 0.83), weight control (α = 0.86), familiarity (α = 0.74), and ethical concern (α = 0.79). Optimistic bias was estimated by asking participants to respond to the following statement ‘How do you think your chances of getting cardiovascular disease (CVD) in the future compare with those of the average adult of your age and sex? Response: 7-point Likert scale (much lower than average - much higher than average).^(36)^ Participants were also asked the same question with reference to type 2 diabetes (T2D) and obesity. The mean score of all three items was used to calculate overall optimistic bias (α = 0.86), a higher score represented a higher level of optimistic bias.

The final section of the survey asked participants how potential outcomes related to genotype-based personalised advice would increase the likelihood of adopting it.^(25)^ Also, items to determine each construct of the TPB related to the adoption of genotype-based dietary and physical activity advice. The direct measures of TPB constructs (attitude (α = 0.88), subjective norms (α = 0.77), PBC (α = 0.81) and intention (α = 0.87) were calculated from the mean score of items for each construct.^(19)^ Belief composites (behavioural, normative, and control beliefs) were calculated as described by Ajzen.^(19)^

### Statistical analysis

Statistical analysis was carried out using IBM SPSS Statistics 26 for Windows (IBM Corp, New York, USA). Measures of centrality and spread are presented as means and SD; categorical data are presented as frequencies and percentages. Comparisons were made between participants who perceived themselves to be normal weight and those who perceived themselves to be overweight or obese. Participants who perceived themselves to be underweight were excluded from analysis (n = 5). Normality of data was assessed using the Shapiro-Wilk test. Baseline continuous measures were not normally distributed (*P* ≥ 0.05) and were compared between groups using a Mann-Whitney *U* test. Categorical variables were compared using a Chi-square Test or when expected counts were less than five, a Fisher’s Exact Test. For *post hoc* analyses, a Bonferroni adjustment was made to correct for multiple comparisons. Stepwise linear multiple regression analysis was conducted to identify the relationship between constructs of the TPB and intention to adopt genotype-based personalised nutrition and to determine the relationship between behavioural beliefs, food choice motives, characteristics and psychological factors, with each construct of the TPB. Each multiple regression was conducted with all participants and separately in those who perceived themselves to be normal weight and those who perceived themselves to be overweight or obese. All tests were two-tailed and considered statistically significant when *P* < 0.05.

## Results

### Participant characteristics

A total of 396 young adults completed the survey; their characteristics are summarised in Table 1. Seventy-six per cent of participants perceived themselves to be normal weight, with 23% overweight or obese, and one per cent underweight. Compared to participants who perceived themselves to be normal weight, participants who perceived themselves to be overweight were more likely to be male (54% v 46%, *P* = 0.001) and reported to be physically active less frequently (3.4 v 4.2 d/week, *P* = 0.001). There was also a significant difference between proportions for how healthy participants considered themselves (*P* < 0.001). Compared to participants who perceived themselves to be overweight or obese, a greater proportion of participants who perceived themselves to be normal weight considered themselves to be very healthy compared to healthy, moderately healthy, or unhealthy. Also, a greater proportion considered themselves to be healthy compared to unhealthy. There was no significant difference between the proportion of participants who perceived themselves to be overweight or obese versus those who perceived themselves to be normal weight, based on their age (*P* = 0.475), ethnicity (*P* = 0.063), country of residence (*P* = 0.179), or highest level of education that they had completed (*P* = 0.317).

**Table 1.** Characteristics for all participants (n = 396), for those who perceive themselves to be normal weight (n = 299) and those who perceive themselves to be overweight or obese (n = 92) data presented as n (%) or mean and SD

Characteristic		Normal weight		Overweight or obese		All participants		P value
		n or mean	% or SD	n or mean	% or SD	n or mean	% or SD	
Gender	*Men*	103	34	50	54	153	39	P = 0.001
	*Women*	196	66	42	46	243	61	
Age	(years)	21	2	21	2	21	2	P = 0.475
Ethnicity	*Asian or Asian British*	29	10	17	19	46	12	P = 0.063
	*Black, Black British, Caribbean, or African*	27	9	8	9	35	9	
	*Mixed or multiple ethnic groups*	18	6	8	9	27	7	
	*White*	214	72	53	58	271	68	
	*Other ethnic group*	11	4	6	7	17	4	
Country of residence	*England*	293	98	87	95	385	97	P = 0.179
	*Wales*	1	0	1	1	2	1	
	*Scotland*	2	1	1	1	3	1	
	*Northern Ireland*	3	1	3	3	6	2	
Education	*Secondary School (GCSE or equivalent)*	9	3	4	4	14	4	P = 0.317
	*Further Education (A Level or equivalent)*	187	63	53	58	243	61	
	*Bachelor’s Degree*	86	29	26	28	112	28	
	*Master’s Degree*	16	5	7	8	24	6	
	*Prefer not to say*	1	0	2	2	3	1	
Health Perception	*Very unhealthy*	3	1	2	2	5	1	P < 0.001
	*Unhealthy*	5	2	9	10	14	4	
	*Moderately unhealthy*	48	16	27	29	77	19	
	*Healthy*	198	66	53	58	253	64	
	*Very healthy*	45	15	1	1	47	12	
Physical activity	(days/week)	4.2	1.9	3.4	1.9	4.0	2.0	P = 0.001
Perceived body image	*Underweight*	0	0	0	0	5	1	
	*Normal weight*	299	100	0	0	299	76	
	*Overweight*	0	0	75	82	75	19	
	*Obese*	0	0	17	19	17	4	

### Psychological factors, motives for food choice, and constructs of the TPB

Mean scores for psychological factors, motives for food choice, and constructs of the TPB were compared between participants who perceived themselves to be normal weight and participants who perceived themselves to be overweight or obese. Participants who perceived themselves to be overweight or obese had a significantly lower internal HLC (3.8 v 4.0, *P* = 0.002), overall optimistic bias (4.2 v 5.2, *P* < 0.001), and optimistic bias for developing CVD (4.3 v 5.0, *P* < 0.001), T2D (4.2 v 5.1, *P* < 0.001) and obesity (4.2 v 5.6, P < 0.001). There were no significant differences between groups for external HLC, food choice motives, or constructs of the TPB (*P* ≥ 0.05). Sensory appeal was the highest-rated food choice motive, followed by price and health. Mean scores for attitude, subjective norms and PBC were positive (Table 2).

**Table 2.** Psychological factors, motives for food choice and constructs of the Theory of Planned Behaviour for all participants (n = 396), and for those who perceive themselves to be normal weight (n = 299) and those who perceive themselves to be overweight or obese (n = 92); data presented as mean and SD

	Normal weight		Overweight or obese		All participants	
	mean	SD	mean	SD	mean	SD
Internal Health locus of control	4.0	0.6	3.8^a^	0.8	4.0	0.7
External Health locus of control	1.7	0.6	1.8	0.7	1.7	0.6
Optimistic bias	5.2	1.3	4.2^a^	1.3	5.0	1.4
CVD	5.0	1.3	4.3^a^	1.4	4.9	1.4
T2D	5.1	1.5	4.2^a^	1.5	4.9	1.5
Obesity	5.6	1.3	4.2^a^	1.8	5.3	1.6
Food choice motives						
Health	3.5	0.7	3.4	0.8	3.5	0.7
Mood	3.3	0.9	3.4	0.8	3.3	0.9
Convenience	3.2	0.8	3.2	1.0	3.1	0.9
Sensory appeal	3.7	1.0	3.6	0.9	3.7	0.8
Natural content	3.1	0.8	2.9	1.1	3.0	1.0
Price	3.5	0.8	3.6	0.8	3.6	0.9
Weight control	2.8	1.0	3.0	1.1	2.8	1.1
Familiarity	2.5	0.9	2.5	0.9	2.5	0.9
Ethical concern	2.2	0.9	2.1	0.9	2.1	0.9
TPB constructs						
Attitude	5.0	1.1	4.9	1.2	5.0	1.1
Subjective norms	4.8	1.1	4.6	1.3	4.7	1.2
Perceived behavioural control	4.8	1.1	4.7	1.0	4.8	1.1
Intention	4.5	1.3	4.5	1.2	4.5	1.3

CVD, cardiovascular disease; T2D, type 2 diabetes; TPB, theory of planned behaviour.

a Significantly different to participants who perceive themselves to have a normal body weight P < 0.05.

### Objective 1: TPB constructs and intention

**Fig. 2.**
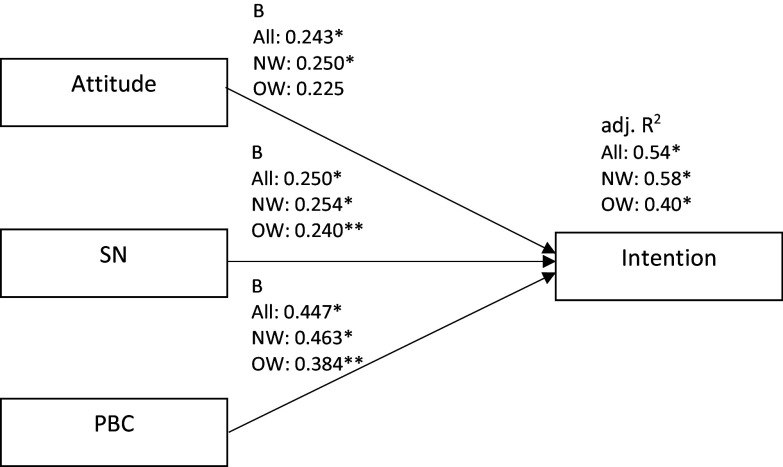
**Objective 1:** Summary of unstandardised regression coefficients and adjusted *R*^*2*^ of constructs of the Theory of Planned Behaviour, for all participants, participants that perceive themselves to be normal weight and participants that perceive themselves to be overweight or obese. B, unstandardised regression coefficient; adj. *R*^*2*^, adjusted *R*^*2*^; SN, subjective norms; PBC, perceived behavioural control; All, all participants (n = 391); NW, participants that perceive themselves to be normal weight (n = 299); OW, participants that perceive themselves to be overweight or obese (n = 92). * P < 0.001; ** P < 0.05.

Multiple regression analysis revealed that attitude, subjective norm, and PBC explained the intention to adopt genotype-based personalised nutrition for all participants (*P* < 0.001, adj. *R*^*2*^ = 0.54; attitude: *B* = 0.24, subjective norm: *B* = 0.25, PBC: *B* = 0.45), those that perceived themselves to be normal weight (*P* < 0.001, adj. *R*^*2*^ = 0.58; attitude: *B* = 0.25, subjective norm: *B* = 0.25, PBC: *B* = 0.46), and those that perceived themselves to be overweight or obese (*P* < 0.001, adj. *R*^*2*^ = 0.40; attitude: *B* = 0.23, subjective norm: *B* = 0.24, PBC: *B* = 0.38). In all models the largest unstandardised regression coefficient was observed for PBC, followed by subjective norm and attitude which was not a significant predictor in the model for participants who perceive themselves to be overweight or obese (Fig. 2, Supplementary Table 6).

### Objective 2

#### Belief composites and TPB constructs

**Fig. 3.**
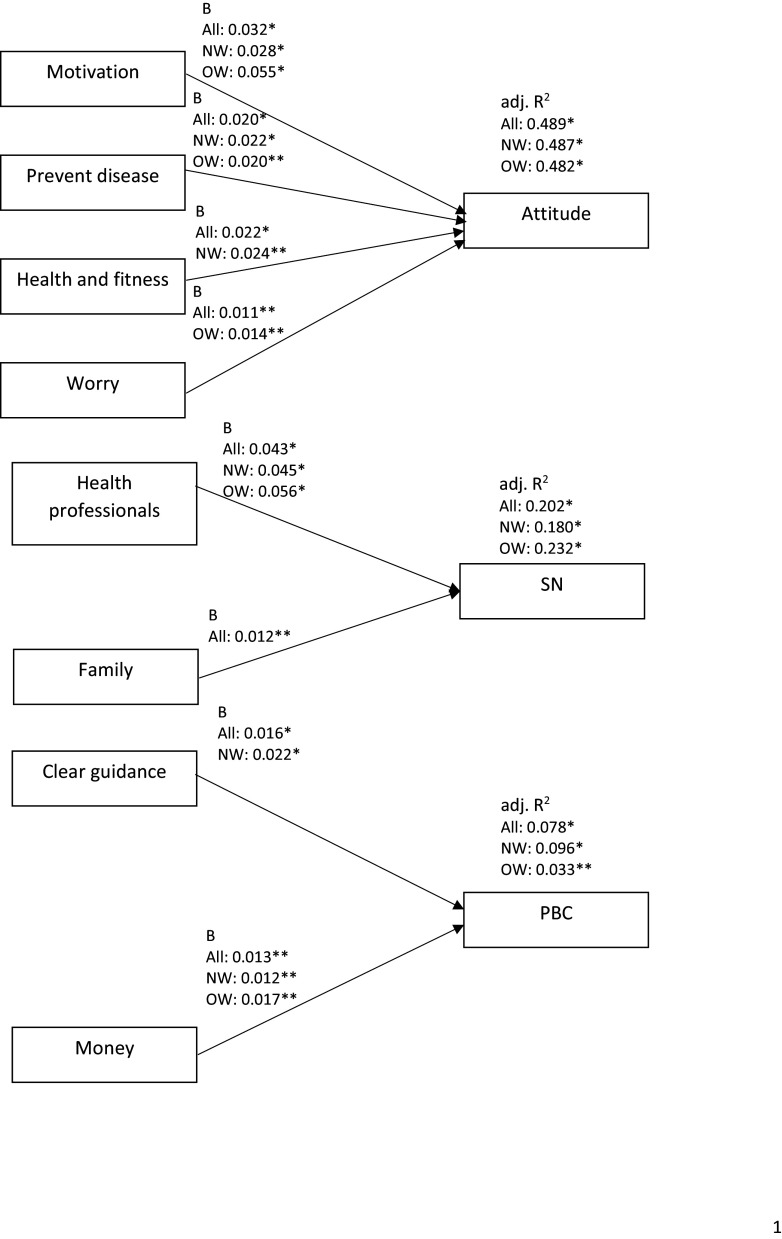
**Objective 2:** Summary of unstandardised regression coefficients and adjusted *R*^*2*^ of constructs of belief composites and Theory of Planned Behaviour constructs, for all participants, participants that perceive themselves to be normal weight and participants that perceive themselves to be overweight or obese. B, unstandardised regression coefficient; adj. *R*^*2*^, adjusted *R*^*2*^; SN, subjective norms; PBC, perceived behavioural control; All, all participants (n = 391); NW, participants that perceive themselves to be normal weight (n = 299); OW, participants that perceive themselves to be overweight or obese (n = 92). * P < 0.001; ** P < 0.05.

Belief composites explained attitude, subjective norms and PBC towards genotype-based personalised advice in all participants (*P* < 0.001, adj. *R*^*2*^ = 0.49; *P* < 0.001, adj. *R*^*2*^ = 0.20; *P* < 0.001, adj. *R*^*2*^ = 0.08), participants that perceived themselves to be normal weight (*P* < 0.001, adj. *R*^*2*^ = 0.49; *P* < 0.001, adj. *R*^*2*^ = 0.18; *P* < 0.001, adj. *R*^*2*^ = 0.10), and participants that perceived themselves to be overweight or obese (*P* < 0.001, adj. *R*^*2*^ = 0.48; *P* < 0.001, adj. *R*^*2*^ = 0.23; *F* = 4.151, *P* = 0.045, adj. *R*^*2*^ = 0.03) (Fig. 3, Supplementary Tables 7–9).

#### Psychological factors, characteristics and TPB constructs

**Fig. 4.**
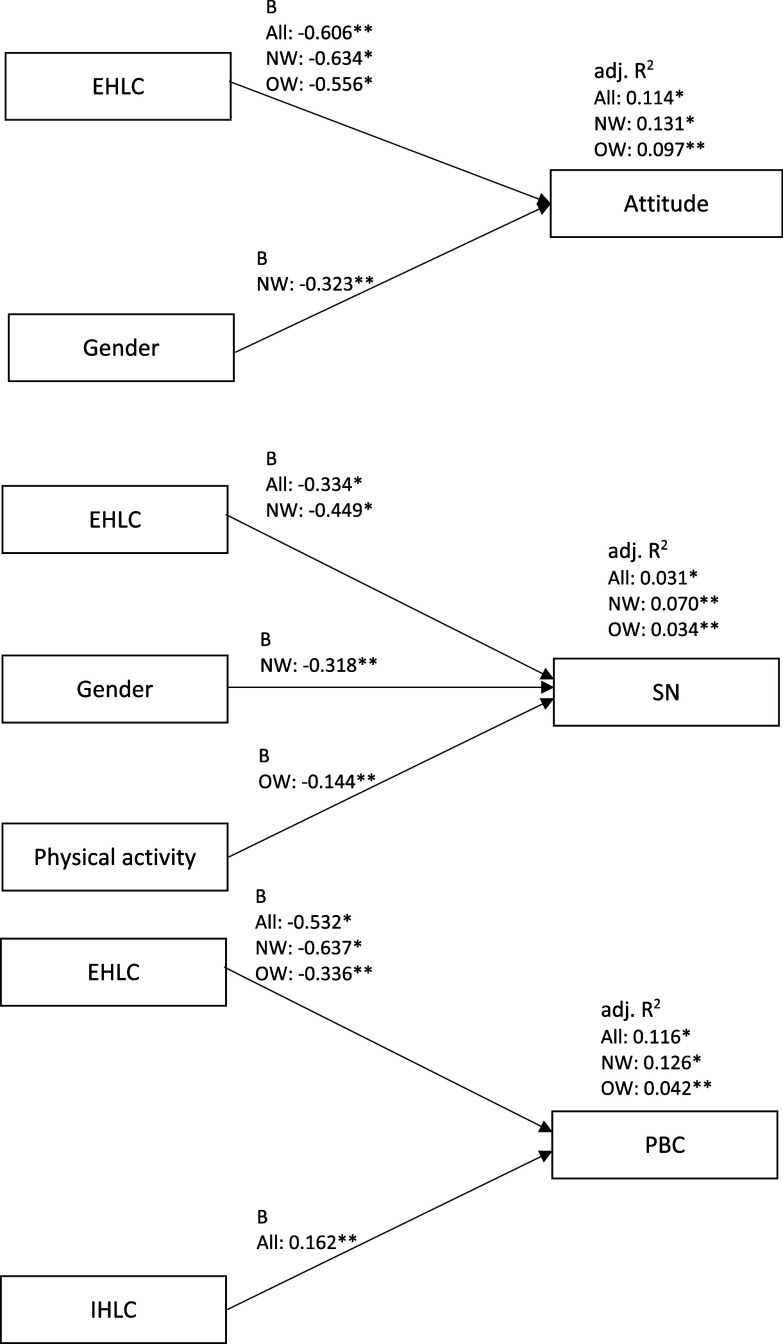
**Objective 2:** Summary of unstandardised regression coefficients and adjusted R^2^ of psychological factors and characteristics for Theory of Planned Behaviour constructs, for all participants, participants that perceive themselves to be normal weight and participants that perceive themselves to be overweight or obese. B, unstandardised regression coefficient; adj. *R*^*2*^, adjusted *R*^*2*^; EHLC, external health locus of control; IHLC, internal health locus of control; SN, subjective norms; PBC, perceived behavioural control; All, all participants (n = 391); NW, participants that perceive themselves to be normal weight (n = 299); OW, participants that perceive themselves to be overweight or obese (n = 92). * P < 0.001; ** P < 0.05.

Psychological factors and characteristics explained attitude, subjective norms, and PBC in all participants (*P* < 0.001, adj. *R*^*2*^ = 0.11; *P* < 0.001, adj. *R*^*2*^ = 0.03; *P* < 0.001, adj. *R*^*2*^ = 0.12), in participants that perceived themselves to be normal weight (*P* < 0.001, adj. *R*^*2*^ = 0.13; *P* < 0.001, adj. *R*^*2*^ = 0.07; *P* < 0.001, adj. *R*^*2*^ = 0.13) and, in participants that perceived themselves to be overweight or obese (*P* = 0.001, adj. *R*^*2*^ = 0.10; *P* = 0.042, adj. *R*^*2*^ = 0.03; *P* = 0.028, adj. *R*^*2*^ = 0.04 ) (Fig. 4, Supplementary Tables 10–12).

#### Food choice motives and TPB constructs

**Fig. 5.**
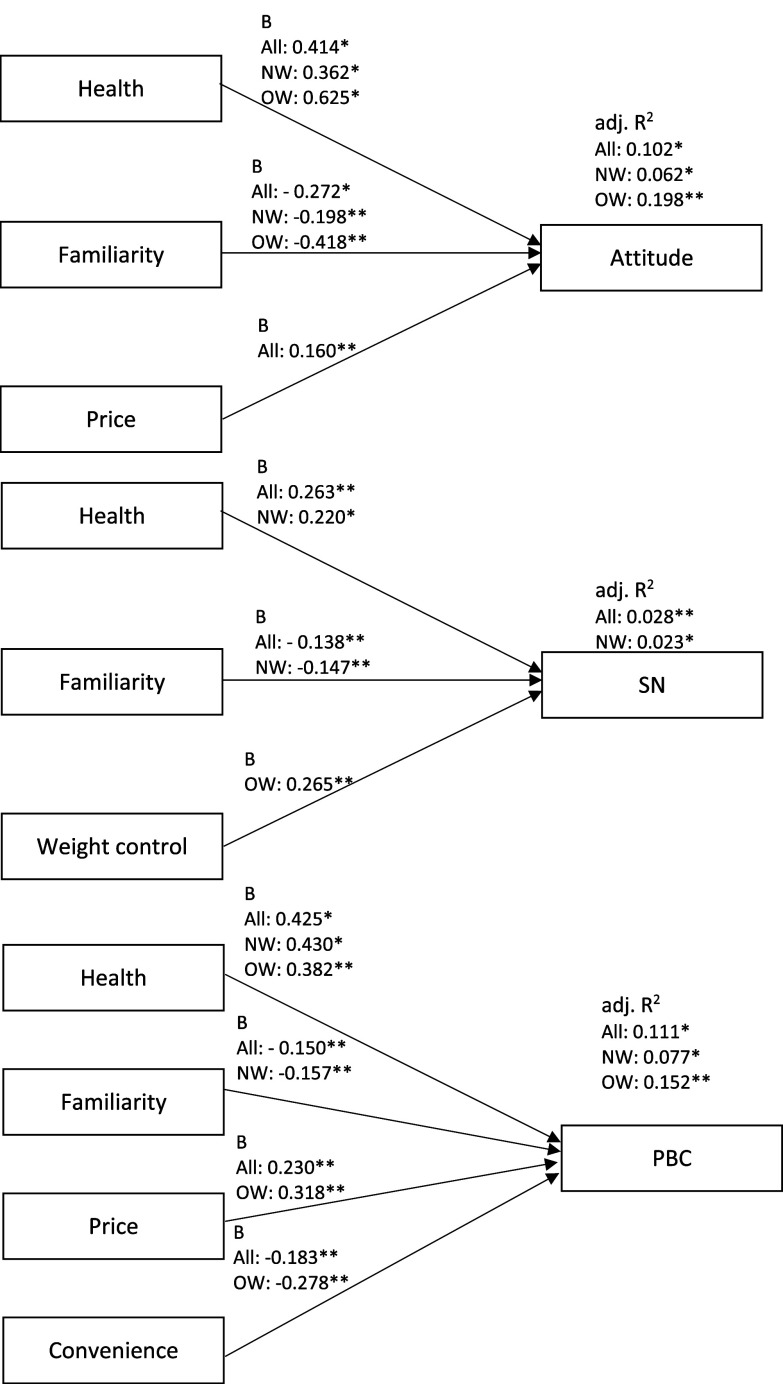
**Objective 2:** Summary of unstandardised regression coefficients and adjusted *R*^2^ of food choice motives for Theory of Planned Behaviour constructs, for all participants, participants that perceive themselves to be normal weight and participants that perceive themselves to be overweight or obese. B, unstandardised regression coefficient; adj. *R*^*2*^, adjusted *R*^*2*^; SN, subjective norms; PBC, perceived behavioural control; All, all participants (n = 391); NW, participants that perceive themselves to be normal weight (n = 299); OW, participants that perceive themselves to be overweight or obese (n = 92). * P < 0.001; ** P < 0.05.

Food choice motives predicted attitude, subjective norms and PBC in all participants (*P* < 0.001, adj. *R*^*2*^ = 0.10; *P* = 0.001, adj. *R*^*2*^ = 0.03; *P* < 0.001, adj. *R*^*2*^ = 0.11), in participants that perceived themselves to be normal weight (*P* < 0.001, adj. *R*^*2*^ = 0.06; *P* = 0.013, adj. *R*^*2*^ = 0.02; *P* < 0.001, adj. *R*^*2*^ = 0.08) and participants that perceived themselves to be overweight or obese (*P* < 0.001, adj. *R*^*2*^ = 0.20; *P* = 0.032, adj. *R*^*2*^ = 0.04; *P* = 0.001, adj. *R*^*2*^ = 0.15) (Fig. 5, Supplementary Tables 13–15).

## Discussion

The aim of this research was to use the TPB as a model to understand the intentions of young adults to adopt genotype-based personalised advice for dietary or physical activity behaviour. On average, young adults have a positive intention to adopt genotype-based advice for dietary and physical activity behaviour, driven by a favourable attitude, a positive perception of social pressure, and perceived ability to perform the behaviour. These findings were consistent in participants who perceived themselves to be normal weight and overweight or obese. To understand the factors that influence the proximal constructs of intention to adopt genotype-based personalised advice, the relationships between belief composites, characteristics and psychological factors, and food choice motives were determined for each construct.

### Attitude towards the behaviour

Behavioural beliefs of ‘motivation to eat healthily and exercise’ and ‘prevent disease’ were significant positive predictors of attitude in all models. ‘To achieve health and fitness goals’ was a significant positive predictor of attitude for all participants and participants that perceive themselves to be normal weight; however, ‘worry about the risk of developing a disease’ was a significant positive predictor for all participants and those that perceived themselves to be overweight or obese. Consequently, when implementing an intervention in young adults who do not perceive themselves to be overweight, highlighting personalised advice as a tool to improve health and fitness may increase uptake, whereas, in a population that deems themselves to be overweight, it may be more effective to highlight the role of personalised advice in disease prevention.

Having an external HLC was a significant negative predictor of attitude towards adoption of genotype-based advice. However, the low mean external HLC score suggested that the majority of participants perceived health to be under their control and scores did not differ significantly between participants based on their body weight perception. Previous research has suggested that internal HLC had a greater capacity to explain variance in diet-related behaviour than external HLC.^(37)^ Internal HLC was significantly positively associated with attitude in the present study (*r =* 0.12) but did not add significantly to the model; furthermore, the negative relationship between external HLC and attitude was stronger (*r =* –0.34). Poínhos *et al.*^(24)^ also reported a stronger association between external, compared to internal, HLC and attitude. Therefore, when investigating personalised nutrition, it appears that external rather than internal HLC has a greater capacity to explain variance in attitude. In the present study, internal HLC was significantly lower in participants who perceived themselves to be overweight or obese compared to those who perceived themselves to be normal weight. Consequently, challenging the perception of young adults that their health is not under their control could improve their attitude towards genotype-based personalised advice. In participants who perceived themselves to be normal weight, men had a significantly less positive attitude towards personalised nutrition than women. Women have been reported to be more conscious of health and demonstrate greater engagement with preventative behaviours.^(38)^ In contrast, men have been reported to have lower adherence to, and belief in, healthy eating recommendations,^(39)^ and are less likely to be willing to have a genetic test.^(26,40)^ In effect, for many aspects of genotype-based personalised nutrition, the advice provided may be more effective if it is personalised by sex.^(41,42)^ Consequently, the findings of the present study are in agreement with the recommendation that interventions to change health behaviours should be developed differently for male and female populations.^(38)^

Food choice motives explained the greatest percentage of variance in the model which included participants who perceived themselves to be overweight or obese (20%) compared to the model which included participants who perceived themselves to be normal weight (6%). In all models, ‘health’ had the largest β-coefficient, and this was greatest in the model of participants who perceived themselves to be overweight or obese. Previous research has highlighted a positive association between the food choice motive of ‘health’ and attitude towards both healthy eating in young adults^(43)^ and attitude towards personalised nutrition in European adults.^(44)^ In the present study, ‘health’ was the third highest-rated food choice motive after ‘sensory appeal’ and ‘price’. ‘Sensory appeal’ and ‘price’ are commonly reported as the highest-rated motives for food choice.^(22,43)^ Consequently, for health motives to be considered in food choice, the food should have sensory appeal and good value. In accordance with previous research, participants who rated ‘familiarity’ as an important motive for food choice had a less favourable attitude towards genotype-based advice.^(44)^ These participants may perceive that genotype-based advice would require them to consume new or different foods from those they normally eat. Eating context has been investigated in previous research and may overlap with the concept of familiarity.^(13,25,45)^ Eating context may be a barrier to the adoption of personalised nutrition, particularly when eating out of the home or with family members.^(13,25,45)^ Therefore, young adults may have a more favourable attitude towards the use of personalised advice if they are assured that food preferences and eating context will be considered in the advice.^(44,45)^

### Subjective norms

In all models ‘health professionals’ were a significant positive predictor of subjective norms. In line with other research, communication of information to young adults about the benefits of personalised dietary and physical activity advice may be most effective when delivered by a health professional.^(23,46)^

Male participants and those who perceived that their health was outside of their own control were less influenced by perceived social pressure to engage with genotype-based personalised dietary or physical activity advice. In participants who perceived themselves to be overweight or obese, a higher level of reported physical activity was associated with lower subjective norms. Since these participants are already engaged in healthy lifestyle behaviours, they may be influenced less by social pressure.

As reported with attitude towards the behaviour, a similar pattern was observed between food choice motives of ‘health’ (significant positive relationship) and ‘familiarity’ (significant negative relationship) with subjective norms. However, in participants who perceived themselves to be overweight or obese, ‘weight control’ was the only significant predictor of subjective norms. Participants who reported ‘weight control’ as a strong motive in their food choices were more influenced by social pressure to engage with genotype-based personalised advice. Previous research has identified the potential for weight loss as a perceived benefit of personalised nutrition^(25)^ as well as being a significant predictor of attitude, intention^(44)^ and acceptance of personalised nutrition advice.^(47)^

### Perceived behavioural control

Control beliefs explained a significant proportion of the variance in PBC in all models, although the percentage of variance explained was trivial (3–10%). ‘Having enough money’ was a significant positive predictor in all models and ‘having confidence in the effectiveness of guidance’ was a positive predictor in the model including all participants and those that perceived themselves to be normal weight. Previous research has reported perceived benefits of personalised advice to have the strongest relationship with attitude, intention,^(24,45,48)^ and acceptance^(47)^ of personalised nutrition. Confidence in the effectiveness of guidance may represent a proportion of what participants would perceive as benefits of personalised advice. Conversely, perceived risk (not measured in the present study) has been reported to have a negative, although less influential, relationship with attitude and intention.^(24,48)^

Participants who perceived greater control over their own health perceived themselves to have greater control over their health-related behaviour. The consistent finding between external HLC and each construct of the TPB once again highlights the importance of communicating how lifestyle behaviour can be as important as genetics in determining the risk of disease^(49)^ and, in terms of increasing PBC, explaining how individuals can achieve or maintain healthy behaviours.

Food choice motives of ‘health’, ‘price’ and ‘familiarity’ influenced participant’s perception of their ability to adopt genotype-based personalised advice to modify their dietary or physical activity behaviour, in a similar manner to attitude and subjective norms. ‘Convenience’ had a significant negative relationship in the model for all participants and those who perceived themselves to be overweight or obese. Participants who rate ‘convenience’ as a strong motive for food choice may perceive the adoption of dietary or physical activity advice to be more challenging. ‘Convenience’ was not identified as a significant factor in the study by Rankin *et al.*^(44)^ and this may be because they only looked at the relationship between food choice motives and attitude and intention to adopt personalised nutrition. The findings of the present study suggest that although there are some consistent patterns between food choice motives and TPB constructs, there are also differences both between constructs and between participants based on their perception of their body weight. An understanding of which factors influence which constructs of the TPB helps to understand the context of how advice should be communicated to young adults. For example, whether it should be phrased to address their appraisal of genotype-based advice (attitude) or their ability to carry out necessary changes in their behaviour (PBC).

### Recommendations

There are some recommendations for the delivery of genotype-based personalised advice to motivate healthy dietary and physical activity behaviour in young adults that appear to be generically applicable to this population. To appreciate the need to meet advice, young adults need to accept the strong effect that these lifestyle behaviours can have on their subsequent health and, importantly, that this is under their control. Advice provided should be delivered in the context of improving health. Food preferences should be considered in the delivery of dietary recommendations and advice should preferably be delivered via a health professional. Advice should detail how to meet dietary and physical activity advice; for example, if a reduction in sodium intake is recommended, advice should explain which foods are high in salt and provide alternative food choices to enable the advice to be met. The findings also suggest that to motivate behaviour change, advice should be tailored based on individual characteristics of young adults. Highlighting the role of genotype-based advice to improve health and fitness is more important for young adults who perceive themselves to be normal weight; whereas, in young adults who perceive themselves to be overweight or obese, advice for disease prevention and weight control would likely be more effective for increasing their intention to adopt advice. Of participants who perceive themselves to be normal weight, young men had a less favourable attitude towards the adoption of genotype-based dietary and physical activity advice and were less influenced by social pressure than young women. Therefore, advice that increases their perceived ability to adopt dietary and physical activity advice may be more effective in increasing their intention to adopt advice. Young adults who believe they are already engaged in healthy lifestyle behaviours or perceive themselves to be normal weight are less likely to perceive a need to adopt genotype-based advice.^(20)^ Optimistic bias has been suggested as a potential barrier to the adoption of personalised nutrition advice, particularly in younger populations.^(13)^ Although optimistic bias did not add significantly to any of the models, it was significantly higher in the participants who perceived themselves to be normal weight and was correlated significantly with participants’ health perception (*r =* 0.33), physical activity (*r =* 0.34), internal HLC (*r =* 0.35), and external HLC (*r =* –0.25). Advice provided to this group should highlight how genes can interact with lifestyle behaviours to affect disease risk, in order to challenge their optimistic bias. Adoption of these recommendations would provide more targeted personalised advice to young adults and as a consequence may result in a more effective intervention to change behaviour.

### Strengths and limitations

The strengths of this study include a specific focus on a young adult population who stand to benefit most from genotype-based personalised advice. The use of the TPB provided a framework to understand the factors that influence the intention to adopt genotype-based personalised advice. The relationship between background factors and subjective norms and PBC in addition to attitude was included and was novel to this research area. However, the study was not without limitations; in several of the regression models, despite being significant, only a small amount of variance was explained by the factors included. Control beliefs were determined from salient beliefs elicited in the pilot study and explained less than 10% of the variance in PBC; in effect, there may be further control factors that make up PBC in this young adult population. Other potential background factors that may have influenced TPB constructs, and intention to adopt genotype-based advice were not included; the most important of which was a measure of risk and benefit. This has been previously well researched with the relatively consistent finding that benefits have a greater influence than risks on intention to adopt genotype-based advice.^(13,24,45,47,48,50)^ Since the risk/benefit relationship with adoption of personal nutrition is relatively well understood, it was not included as a measure in the present study; however, it may account for a proportion of the unexplained variance in the models.

### Conclusions

In conclusion, the current study provides support for the use of the TPB in understanding the intention of young adults to adopt genotype-based advice for dietary and physical activity behaviour. Background factors including belief composites, HLC, gender, physical activity, and food choice motives of ‘health’, ‘price’, ‘familiarity’, ‘weight control’, and ‘convenience’ interact with TPB constructs. In addition to perceived body weight, these background factors should be utilised to inform the delivery of advice in behaviour change interventions that seek to use genotype-based personalised advice in young adult populations. Finally, the recommendations for the use of genotype-based dietary and physical activity advice in young adults, based on the findings of the present study, need to be evaluated in a genotype-based personalised nutrition intervention study to change dietary behaviour.

## Supporting information

King et al. supplementary material King et al. supplementary material

## Abbreviations

BMI: body mass index
HLC: health locus of control
NCD: non-communicable diseases
PBC: perceived behavioural control
T2D: type 2 diabetes
TPB: Theory of Planned Behaviour

## Supplementary material

The supplementary material for this article can be found at https://doi.org/10.1017/jns.2024.50
