# Supplementary material for: Factors influencing the intention of young adults to adopt genotype-based personalised advice on diet and physical activity according to perceived weight status
Source: J Nutr Sci. 2024 Sep 30;13:e54. doi: 10.1017/jns.2024.50 (PMC11514639; doi:10.1017/jns.2024.50)
Supplement: King et al. supplementary material [file S2048679024000508sup001.docx]

#### **Supplementary material:**

#### *Usability of the survey*

The majority of participants reported that the survey took them 15-20 minutes to complete (n = 25; 72%); the time reported ranged from 10 – 32 minutes. All participants responded positively to the question to determine if they understood the definition of personalised nutrition; one participant suggested that additional information be provided. Four participants commented on finding the switching of Likert scales from positive to negative for some questions and negative to positive for others confusing. Literature suggests that the use of reverse-scored items in questionnaires is debatable; although it may reduce response set bias, it increases cognitive processing demands and may affect psychometric properties of the question (Tsang et al., 2017). Consequently, reverse-scoring was not used in the final survey.

#### *Internal consistency*

Internal consistency is a measure of homogeneity or reliability among items used to measure the same construct and was assessed using α (Tavakol & Dennick, 2011). Values for each construct are presented in Table 1. An α > 0.7 has been suggested to indicate adequate internal consistency (Tavakol & Dennick, 2011). This was met for all constructs except subjective norms. When item 4 was deleted, the α increased but remained below the 0.7 cut-off, α > 0.6 has also been described in some literature as acceptable (Taber, 2018). Since reducing the number of items would reduce the α and the value was close to acceptability, the remaining items to measure subjective norms were retained and internal consistency was reassessed in the final survey with a larger sample size.

**Table 1.** Assessment of internal consistency.

| Construct | Cronbach’s alpha |
| --- | --- |
| Attitudes | 0.755 |
| Subjective norms | 0.557 (0.567)* |
| Perceived behavioural control | 0.723 |
| Intention | 0.735 |

*Change in Cronbach’s alpha following deletion of item 4 for subjective norms.

#### *Discriminant validity*

Discriminant validity determines whether constructs that are not theoretically related are unrelated to other constructs (Campbell & Fiske, 1959). Discriminant validity was assessed using heterotrait-monotrait ratio of correlations (HTMT) (Henseler et al., 2015). The results of HTMT for each pair of constructs are presented in Table 2. The HTMT ratios were all below 0.90 except for attitudes and subjective norms (0.93). Hensler et al. (2015) suggests a HTMT ratio greater than 0.9 signifies a problem with discriminant validity between these constructs. To retain the constructs that suggest a problem with discriminant validity, Henseler et al. (2015) recommend eliminating items that have low correlations with items measuring the same construct. Removal of the fourth item measuring subjective norms increased the monotrait correlation for subjective norms from 0.239 to 0.305. The HTMT ratio for attitudes and subjective norms was subsequently reduced to 0.823 meeting the criteria for discriminant validity. Therefore, item 4 for subjective norms was removed from the final survey.

**Table 2.** Assessment of discriminant validity.

|  | Attitudes | Subjective norms | Perceived behavioural control |
| --- | --- | --- | --- |
| Attitudes |  |  |  |
| Subjective norms | 0.93 (0.823)* |  |  |
| Perceived behavioural control | 0.85 | 0.305 |  |
| Intention | 0.80 | 0.53 | 0.71 |

*Change in HTMT ratio following deletion of item 4 for subjective norms.

#### *Constructing items to measure control beliefs*

Pilot study items were used to elicit salient outcomes and experiences, normative referents, and control factors related to the adoption of genotype-based advice to modify dietary or physical activity behaviour in a young adult population. A content analysis of participant responses provided a list of modal salient outcomes, referents and control factors. These were used to construct items to assess: i) the strength of behavioural beliefs and evaluation of the outcome experience; ii) normative referents and motivation to comply; and iii) control beliefs and power of control factors.

| **Salient belief** | **Item** |
| --- | --- |
| **Behavioural outcomes and experiences**  Achieve health and fitness goals  Provide motivation  Restrictive  Effort and time to make changes  Prevent disease  Concern about risk  Cost | **Behavioural belief strength** (likely-unlikely)  genotype-based advice to modify dietary or physical activity behaviour will help me to achieve health and fitness goals.  genotype-based advice to modify dietary or physical activity behaviour will provide me with motivation to eat healthily and exercise.  genotype-based advice to modify dietary or physical activity behaviour will restrict my food choices.  genotype-based advice to modify dietary or physical activity behaviour will take effort and time to make changes.  genotype-based advice to modify dietary or physical activity behaviour will help me to prevent disease.  genotype-based advice to modify dietary or physical activity behaviour will cause me to worry about the risk of developing a disease.  genotype-based advice to modify dietary or physical activity behaviour will be expensive.  **Outcome evaluation** (good - bad)  For me achieving health and fitness goals is  For me to prevent the development of disease is  For me to be motivated to eat healthily and exercise is  For me restriction of my food choices is  For me to take effort and time to make changes is  For me the expense of genotype-based advice is  For me to worry about the risk of developing a disease is |
| **Normative referents**  Friends  Family  Influencers  Health professionals | **Normative beliefs** (likely-unlikely)  My friends would think I should use genotype-based advice to modify dietary or physical activity behaviour.  My family would think I should use genotype-based advice to modify dietary or physical activity behaviour.  Influencers and people I follow on social media would think I should use genotype-based advice to modify dietary or physical activity behaviour.  Health professionals would think I should use genotype-based advice to modify dietary or physical activity behaviour.  **Motivation to comply** (agree-disagree)  When it comes to matters of health, I want to do what my friends think I should do.  When it comes to matters of health, I want to do what my family think I should do.  When it comes to matters of health, I want to do what influencers and people I follow on social media think I should do.  When it comes to matters of health, I want to do what health professionals think I should do. |
| **Control factors**  Time  Clear guidance  Confidence in advice  Cost | **Control belief** (rarely - frequently)  How often does lack of time prevent you from eating healthily and or exercising?  How often does lack of clear guidance prevent you from eating healthily and or exercising?  How often does lack of confidence in effectiveness of guidance prevent you from eating healthily and or exercising?  How often does lack of money prevent you from eating healthily and or exercising?  **Power of control factor** (agree-disagree)  Having enough time would enable me to adopt genotype-based advice to modify dietary or physical activity behaviour.  Having enough money would enable me to adopt genotype-based advice to modify dietary or physical activity behaviour.  Having confidence in the effectiveness of guidance would enable me to adopt genotype-based advice to modify dietary or physical activity behaviour.  Having clear guidance would enable me to adopt genotype-based advice to modify dietary or physical activity behaviour. |

**Table 3.**  Modal salient beliefs from content analysis and items added to final questionnaire.

**Table 6** Multiple regression results for intention to adopt genotype-based personalised nutrition from TPB constructs for all participants, participants that perceived themselves normal weight, and participants that perceived themselves overweight or obese.

| Intention | *B* | 95% CI for *B* | SE *B* | *β* | *R^2^* | Δ *R^2^* |
| --- | --- | --- | --- | --- | --- | --- |
| All  Constant  PBC  SN  Attitude | -.025  .447*  .250*  .243* | -.448 .398  .339 .555  .152 .347  .135 .352 | .215  .055  .050  .055 | .390*  .234*  .223* | 0.546 | 0.542* |
| NW  Constant  PBC  SN  Attitude | -.189  .463*  .254*  .250* | -.655 .278  .339 .588  .134 .374  .125 .374 | .237  .063  .061  .063 | .407*  .227*  .224* | 0.584 | 0.580* |
| OW  Constant  PBC  SN  Attitude | .514  .384**  .240**  .225 | -.508 1.537  .148 .620  .061 .419  -.002 .452 | .514  .119  .090  .114 | .323**  .258**  .219 | 0.420 | 0.400* |

Model: Stepwise method; B: unstandardized regression coefficient; CI: confidence interval; SE B: standard error of the coefficient; β: standardised coefficient; *R^2^*: coefficient of determination; Δ *R^2^*: adjusted *R^2^*; SN: subjective norms; PBC: perceived behavioural control, All: all participants (n = 391); NW: participants that perceive themselves to be normal weight (n = 299); OW: participants that perceive themselves to be overweight or obese (n = 92). * *p* < 0.001.

**Table 7** Multiple regression results for attitude towards genotype-based personalised nutrition from behavioural beliefs for all participants, participants that perceived themselves normal weight, and participants that perceived themselves overweight or obese.

| Attitude | B | 95% CI for B | SE B | β | *R^2^* | *Δ R^2^* |
| --- | --- | --- | --- | --- | --- | --- |
| All  (Constant)  Motivation  Prevent disease  Health and fitness  Worry | 2.627*  .032*  .020*  .022*  .011** | 2.354 2.900  .020 .044  .010 .031  .010 .034  .002 .020 | .139  .006  .005  .006  .004 | .336*  .199*  .231*  .092** | .495 | .489* |
| NW  (Constant)  Motivation  Prevent disease  Health and fitness  Worry | 2.608*  .028*  .022*  .024**  .014** | 2.295 2.922  .014 .042  .010 .034  .010 .038  .003 .024 | .159  .007  .006  .007  .005 | .298*  .212*  .248**  .111** | .494 | .487* |
| OW  (Constant)  Motivation  Prevent disease | 2.804*  .055*  .020** | 2.314 3.294  .035 .075  .001 .039 | .247  .010  .010 | .551*  .207** | 0.494 | 0.482* |

Model: Stepwise method; B: unstandardized regression coefficient; CI: confidence interval; SE B: standard error of the coefficient; β: standardised coefficient; *R^2^*: coefficient of determination; Δ *R^2^*: adjusted *R^2^*. All: all participants (n = 391); NW: participants that perceive themselves to be normal weight (n = 299); OW: participants that perceive themselves to be overweight or obese (n = 92) * *p*< 0.001, ** *p* < 0.05.

**Table 8** Multiple regression results for subjective norms from normative beliefs for all participants, participants that perceived themselves normal weight and participants that perceived themselves overweight or obese.

| SN | B | 95% CI for B | SE B | β | *R^2^* | Δ *R^2^* |
| --- | --- | --- | --- | --- | --- | --- |
| All  Constant  Health professionals  Family | 3.571*  .043*  .012** | 3.320 3.821  .033 .054  .000 .024 | .127  .005  .006 | .401*  .102** | .206 | .202* |
| NW  Constant  Health professionals | 3.734*  .045* | 3.456 4.013  .034 .056 | .141  .006 | .427* | .183 | .180* |
| OW  Constant  Health professionals | 3.310*  .056* | 2.786 3.834  .035 .077 | .264  .011 | .491* | 0.241 | 0.232* |

Model: Stepwise method; B: unstandardized regression coefficient; CI: confidence interval; SE B: standard error of the coefficient; β: standardised coefficient; *R^2^*: coefficient of determination; Δ *R^2^*: adjusted *R^2^*. All: all participants (n = 391); NW: participants that perceive themselves to be normal weight (n = 299); OW: participants that perceive themselves to be overweight or obese (n = 92) * *p*< 0.001, ** *p* < 0.05.

**Table 9** Multiple regression results for perceived behavioural control from control beliefs for all participants, participants that perceived themselves normal weight and participants that perceived themselves overweight or obese.

| PBC | B | 95% CI for B | SE B | β | *R^2^* | *Δ R^2^* |
| --- | --- | --- | --- | --- | --- | --- |
| All  Constant  Clear guidance  Money | 4.186*  .016**  .013** | 3.955 4.417  .006 .027  .004 .023 | .117  .005  .005 | .176**  .156** | .083 | .078* |
| NW  Constant  Clear guidance  Money | 4.142*  .022*  .012** | 3.877 4.407  .010 .034  .001 .023 | .135  .006  .006 | .227*  .137** | .103 | .096* |
| OW  Constant  Money | 4.343*  .017** | 3.946 4.740  .000 .033 | .200  .008 | .210 | 0.044 | 0.033** |

Model: Stepwise method; PBC: perceived behavioural control; B: unstandardized regression coefficient; CI: confidence interval; SE B: standard error of the coefficient; β: standardised coefficient; *R^2^*: coefficient of determination; Δ *R^2^*: adjusted *R^2^*. All: all participants (n = 391); NW: participants that perceive themselves to be normal weight (n = 299); OW: participants that perceive themselves to be overweight or obese (n = 92). * *p* < 0.001, ** *p* < 0.05.

**Table 10** Multiple regression results for attitude towards genotype-based personalised nutrition from psychological factors and characteristics for all participants, participants that perceived themselves normal weight and participants that perceived themselves overweight or obese.

| Attitude | B | 95% CI for B | SE B | β | *R^2^* | Δ *R^2^* |
| --- | --- | --- | --- | --- | --- | --- |
| All  Constant  EHLC | 6.007**  -.606** | 5.705 6.309  -.773 -.440 | .154  .085 | -.341** | .116 | .114* |
| NW  Constant  EHLC  Gender | 6.171*  -.634*  -.323** | 5.813 6.529  -.827 -.442  -.578 -.069 | .182  .098  .129 | -.350*  -.135** | .137 | .131** |
| OW  Constant  EHLC | 5.895*  -.556** | 5.254 6.536  -.892 -.219 | .323  .169 | -.327** | 0.107 | 0.097** |

Model: Stepwise method; B: unstandardized regression coefficient; CI: confidence interval; SE B: standard error of the coefficient; β: standardised coefficient; *R^2^*: coefficient of determination; Δ *R^2^*: adjusted *R^2^*. All: all participants (n = 391); NW: participants that perceive themselves to be normal weight (n = 299); OW: participants that perceive themselves to be overweight or obese (n = 92). * *p*< 0.001, ** *p* < 0.05.

**Table 11** Multiple regression results for subjective norms from psychological factors and characteristics for all participants, participants that perceived themselves normal weight and participants that perceived themselves overweight or obese.

| SN | B | 95% CI for B | SE B | β | *R^2^* | Δ *R^2^* |
| --- | --- | --- | --- | --- | --- | --- |
| All  Constant  EHLC | 5.298*  -.334* | 4.975 5.622  -.513 -.156 | .165  .091 | -.183* | .034 | .031* |
| NW  Constant  EHLC  Gender | 5.638*  -.449*  -.318** | 5.269 6.007  -.647 -.250  -.581 -.055 | .188  .101  .133 | -.249*  -.133** | .077 | .070** |
| OW  Constant  Physical activity | 5.068*  -.144** | 4.527 5.609  -.283 -.005 | .272  .070 | -.212** | 0.045 | 0.034** |

Model: Stepwise method; B: unstandardized regression coefficient; CI: confidence interval; SE B: standard error of the coefficient; β: standardised coefficient; *R^2^*: coefficient of determination; Δ *R^2^*: adjusted *R^2^*. All: all participants (n = 391); NW: participants that perceive themselves to be normal weight (n = 299); OW: participants that perceive themselves to be overweight or obese (n = 92). * *p*< 0.001, ** *p* < 0.05.

**Table 12** Multiple regression results for perceived behavioural control from psychological factors and characteristics for all participants, participants that perceived themselves normal weight and participants that perceived themselves overweight or obese.

| PBC | B | 95% CI for B | SE B | β | *R^2^* | Δ *R^2^* |
| --- | --- | --- | --- | --- | --- | --- |
| All  Constant  EHLC  IHLC | 5.056*  -.532*  .162** | 4.348 5.764  -.693 -.370  .013 .311 | .360  .082  .076 | -.313*  .103** | .120 | .116* |
| NW  Constant  EHLC | 5.901*  -.637* | 5.564 6.238  -.826 -.448 | .171  .096 | -.359* | .129 | .126* |
| OW  Constant  EHLC | 5.292*  -.336** | 4.721 5.863  -.636 -.036 | .287  .151 | -.229** | 0.052 | 0.042** |

Model: Stepwise method; PBC: perceived behavioural control; B: unstandardized regression coefficient; CI: confidence interval; SE B: standard error of the coefficient; β: standardised coefficient; *R^2^*: coefficient of determination; Δ *R^2^*: adjusted *R^2^*. All: all participants (n = 391); NW: participants that perceive themselves to be normal weight (n = 299); OW: participants that perceive themselves to be overweight or obese (n = 92). * *p* < 0.001, ** *p* < 0.05.

**Table 13** Multiple regression results for attitude towards genotype-based personalised nutrition from food choice motives for all participants, participants that perceived themselves normal weight and participants that perceived themselves overweight or obese.

| Attitude | B | 95% CI for B | SE B | β | *R^2^* | Δ *R^2^* |
| --- | --- | --- | --- | --- | --- | --- |
| All  Constant  Health  Familiarity  Price | 3.659  .414*  -.272*  .160** | 2.982 4.335  .264 .563  -.397 -.148  .029 .292 | .344  .076  .063  .067 | .264*  -.213*  .119** | .109 | .102* |
| NW  Constant  Health  Familiarity | 4.234*  .362*  -.198** | 3.522 4.947  .181 .543  -.338 -.058 | .362  .092  .071 | .222*  -.157** | .068 | .062* |
| OW  Constant  Health  Familiarity | 3.840*  .625*  -.418** | 2.853 4.827  .349 .900  -.674 -.161 | .497  .139  .129 | .442*  -.317** | .215 | .198** |

Model: Stepwise method; B: unstandardized regression coefficient; CI: confidence interval; SE B: standard error of the coefficient; β: standardised coefficient; *R^2^*: coefficient of determination; Δ *R^2^*: adjusted *R^2^*. All: all participants (n = 391); NW: participants that perceive themselves to be normal weight (n = 299); OW: participants that perceive themselves to be overweight or obese (n = 92) * *p*< 0.001, ** *p* < 0.05.

**Table 14** Multiple regression results for subjective norms from food choice motives for all participants, participants that perceived themselves normal weight and participants that perceived themselves overweight or obese.

| SN | B | 95% CI for B | SE B | β | *R^2^* | Δ *R^2^* |
| --- | --- | --- | --- | --- | --- | --- |
| All  Constant  Health  Familiarity | 4.164*  .263**  -.138** | 3.550 4.778  .104 .422  -.268 -.008 | .312  .081  .066 | .164**  -.105** | .033 | .028** |
| NW  Constant  Health  Familiarity | 4.379*  .220**  -.147** | 3.654 5.105  .036 .404  -.289 -.004 | .369  .094  .072 | .135**  -.116** | .029 | .023** |
| OW  Constant  Weight control | 3.783*  .265** | 3.015 4.551  .023 .507 | .387  .122 | .224** | .050 | .040** |

Model: Stepwise method; SN: subjective norms; B: unstandardized regression coefficient; CI: confidence interval; SE B: standard error of the coefficient; β: standardised coefficient; *R^2^*: coefficient of determination; Δ *R^2^*: adjusted *R^2^*. All: all participants (n = 391); NW: participants that perceive themselves to be normal weight (n = 299); OW: participants that perceive themselves to be overweight or obese (n = 92). * *p* < 0.001, ** *p* < 0.05.

**Table 15** Multiple regression results for perceived behavioural control from food choice motives for all participants, participants that perceived themselves normal weight and participants that perceived themselves overweight or obese.

| PBC | B | 95% CI for B | SE B | β | *R^2^* | Δ *R^2^* |
| --- | --- | --- | --- | --- | --- | --- |
| All  Constant  Health  Familiarity  Price  Convenience | 3.474*  .425*  -.150**  .230**  -.183** | 2.820 4.128  .282 .568  -.275 -.026  .097 .363  -.317 -.049 | .333  .073  .063  .068  .068 | .284*  -.123**  .178**  -.148** | .120 | .111* |
| NW  Constant  Health  Familiarity | 3.724*  .430*  -.157** | 3.031 4.417  .255 .606  -.293 -.020 | .352  .089  .069 | .269*  -.126** | .083 | .077* |
| OW  Constant  Health  Convenience  Price | 3.138*  .382**  -.278**  .318** | 2.011 4.265  .140 .624  -.482 -.074  .081 .555 | .567  .122  .103  .119 | .313**  -.280**  .269** | .180 | .152** |

Model: Stepwise method; PBC: perceived behavioural control; B: unstandardized regression coefficient; CI: confidence interval; SE B: standard error of the coefficient; β: standardised coefficient; *R^2^*: coefficient of determination; Δ *R^2^*: adjusted *R^2^*. All: all participants (n = 391); NW: participants that perceive themselves to be normal weight (n = 299); OW: participants that perceive themselves to be overweight or obese (n = 92) * *p* < 0.001, ** *p* < 0.05.
